# Conversion of Sensitive Data to the Observational Medical Outcomes Partnership Common Data Model: Protocol for the Development and Use of Carrot

**DOI:** 10.2196/60917

**Published:** 2025-04-02

**Authors:** Samuel Cox, Erum Masood, Vasiliki Panagi, Calum Macdonald, Gordon Milligan, Scott Horban, Roberto Santos, Chris Hall, Daniel Lea, Simon Tarr, Shahzad Mumtaz, Emeka Akashili, Andy Rae, Esmond Urwin, Christian Cole, Aziz Sheikh, Emily Jefferson, Philip Roy Quinlan

**Affiliations:** 1 Digital Research Service University of Nottingham Nottingham United Kingdom; 2 Health Informatics Centre University of Dundee Dundee United Kingdom; 3 University of Edinburgh Edinburgh United Kingdom; 4 NIHR Nottingham Biomedical Research Centre School of Medicine University of Nottingham Nottingham United Kingdom; 5 Nuffield Department of Primary Care Health Sciences University of Oxford Oxford United Kingdom

**Keywords:** data standardization, OMOP, Observational Medical Outcomes Partnership, ETL, extract, transform, and load, data discovery, transparency, Carrot tool, common data model, data standard, health care, data model, data protection, data privacy, open-source

## Abstract

**Background:**

The use of data standards is low across the health care system, and converting data to a common data model (CDM) is usually required to undertake international research. One such model is the Observational Medical Outcomes Partnership (OMOP) CDM. It has gained substantial traction across researchers and those who have developed data platforms. The Observational Health Care Data Sciences and Informatics (OHDSI) partnership manages OMOP and provides many open-source tools to assist in converting data to the OMOP CDM. The challenge, however, is in the skills, knowledge, know-how, and capacity within teams to convert their data to OMOP. The European Health Care Data Evidence Network provided funds to allow data owners to bring in external resources to do the required conversions. The Carrot software (University of Nottingham) is a new set of open-source tools designed to help address these challenges while not requiring data access by external resources.

**Objective:**

The use of data protection rules is increasing, and privacy by design is a core principle under the European and UK legislations related to data protection. Our aims for the Carrot software were to have a standardized mechanism for managing the data curation process, capturing the rules used to convert the data, and creating a platform that can reuse rules across projects to drive standardization of process and improve the speed without compromising on quality. Most importantly, we aimed to deliver this design-by-privacy approach without requiring data access to those creating the rules.

**Methods:**

The software was developed using Agile approaches by both software engineers and data engineers, who would ultimately use the system. Experts in OMOP were used to ensure the approaches were correct. An incremental release program was initiated to ensure we delivered continuous progress.

**Results:**

Carrot has been delivered and used on a project called COVID-Curated and Open Analysis and Research Platform (CO-CONNECT) to assist in the process of allowing datasets to be discovered via a federated platform. It has been used to create over 45,000 rules, and over 5 million patient records have been converted. This has been achieved while maintaining our principle of not allowing access to the underlying data by the team creating the rules. It has also facilitated the reuse of existing rules, with most rules being reused rather than manually curated.

**Conclusions:**

Carrot has demonstrated how it can be used alongside existing OHDSI tools with a focus on the mapping stage. The COVID-Curated and Open Analysis and Research Platform project successfully managed to reuse rules across datasets. The approach is valid and brings the benefits expected, with future work continuing to optimize the generation of rules.

**International Registered Report Identifier (IRRID):**

RR1-10.2196/60917

## Introduction

In health research, there are dozens of standards that can be used to represent data, and a recurring challenge surrounding the adoption of existing standards versus the creation of yet more. The retrospective adoption of standards is conceptually simple but is much harder to implement in datasets that are still actively collecting new data. Clearly, if everyone adopted a single standard from the start of all data capture, then such a problem would not exist; the reality of course is very different and data handling practices still vary considerably between research projects and health care organizations. The Observational Medical Outcomes Partnership (OMOP) Common Data Model (CDM) [1,2] is an international open community standard to standardize the schema and contents of the data by using a standardized vocabulary and medical terminology into clinical domains of OMOP CDM. As a concept, the ability to map between a research ontology, such as the International Classification of Disease (ICD) and Systematized Nomenclature of Medicine Clinical Terms (SNOMED-CT), with a single representation within the vocabulary is of immense value [3]. For an organization with data that are not in OMOP, there will always be an effect required to convert the data from the source standard to the OMOP CDM.

The Observational Health Care Data Sciences and Informatics (OHDSI) [4] program collates a suite of open-source tools [5,6] that can assist in the process of the extract, transform, and load (ETL) stages to convert data from the original format to OMOP. There are also tools that can assist in finding the most appropriate concept to use from the vocabulary using a similarity search [7]. Therefore, tools and options do exist that can assist in the conversion of data to the OMOP common standard. However, if the organization either does not have the knowledge or the capacity to undertake the conversion, the “final mile challenge” can be significant. White Rabbit [6] can profile the data and produce a metadata extract. Rabbit-in-a-Hat [5] can allow a data engineer to capture the required transformations in note form. What is missing, though, is an end-to-end process and the conversion of the outputs into actionable code. At present, an individual would most likely generate an SQL command or software script to convert the data based on the interpretations of the Rabbit-in-a-Hat output. This would require local expertise to create. Organizations may have a desire to adopt or curate data to a single standard, but the local capabilities do not always exist.

The European Health Care Data and Evidence Network (EHDEN) [8] is a Horizon IMI program established to help with this gap that funds approved organizations to work with data owners to convert their datasets to OMOP. This is a competitive process that will support datasets that can bring the most value to the wider OHDSI [4] consortium for data sharing. Not all datasets will qualify for support from EHDEN given its finite resources (it clearly cannot support every project seeking to transform to OMOP), and as so, it has eligibility criteria on the periodic calls it makes for support [9].

Most ETL processes work based on having access to the data at the time of designing the conversion, and indeed, the OHDSI tools run on the source data; allow notes to be curated for how data should be converted; and typically require manual development of scripts to convert data from source to OMOP. This approach relies on a team having access to the data, which may not for privacy reasons be desirable in relation to health care and sensitive research datasets. Some organizations may be cautious in allowing external organizations with OMOP knowledge access to core systems and data to undertake the ETL process. In many cases, this will involve setting up legal agreements such as data sharing or confidentiality agreements or generating pseudonymized versions of the data, which are often lengthy and complex tasks.

While, OMOP offers significant power and opportunity as a unifying standard, allowing differences in terminology to be mapped to a single standard, it does suffer from the challenge that curating data to OMOP can still be quite an art form with different curators taking different approaches. The vocabulary used within the OMOP CDM can be downloaded from the website Athena [10]. There is not a single vocabulary set to use, and therefore, the curators may have preferred vocabularies to represent the data. The vocabulary is also periodically updated resulting in a concept changing domains between releases, such as from being in the Observation domain to the Clinical Occurrence domain. The consequence is that someone curating the data today may make a different decision in the future because the vocabulary has changed. The vocabularies in Athena are in English, and therefore, a base assumption of Carrot is that English is the language in use.

The FAIR (Findable, Accessible, Interoperable, and Reusable) principles [11] are a globally accepted set of standards to promote best practices in datasets. CDMs can aid datasets to become interoperable, as they standardize all data to a single model. However, in the quest for interoperability, it is important that the provenance of the data is not lost when it is converted, as there is a risk of an illusion being created because data from multiple organizations are the same. Therefore, while converting data to OMOP can assist in making data FAIR, as it simplifies many of those challenges, it cannot be at the expense of understanding how data have been converted to OMOP.

There are many existing mechanisms to map and covert data [12-14] to the OMOP CDM, and those processes are well established. This paper does not seek to suggest the work presented here is better than any existing processes and protocols. The processes described here were in response to a specific set of constraints, such as limited availability of technical staff members, a desire to create a FAIR resource as a consequence, and most importantly that access to data was not available in most circumstances.

In this paper, we introduce Carrot, a software tool that aims to address these difficulties—namely the governance requirements around bringing OMOP expertise to the datasets, variations between individual curators within the OMOP framework, and the gap between current OHDSI tooling—while also automating as much of the process as possible. Carrot enables curators to map data to OMOP without needing access to the underlying data. This allows a central team of OMOP experts to undertake data curation of many datasets to enhance quality control and standardization while fulfilling a desire to ensure this central team never has access to data that would be in the scope of General Data Protection Regulation (GDPR). Separating the specialized OMOP knowledge from the application of the ETL process allows reduced and shortened governance work, saving data owners time and money in converting their datasets.

Similarly, Carrot provides tooling to guide curators toward standardization of their used OMOP terms wherever possible and automates much of the ETL process. In solving these challenges, Carrot brings reproducibility and transparency to the OMOP data curation process to assist datasets in meeting the increasing FAIR requirements.

## Methods

### Development Principles

The Carrot tools were developed using the Scrum Agile methodology [15] to deliver minimum viable products of each component followed by iterative development to expand the functionality over time. The team consisted of data engineers, OMOP experts, research software engineers, clinical academics, and patient and public representatives. All tools were made available via an MIT license [16] and hosted in GitHub repositories [17,18].

### Privacy-by-Design Principles

The data held by an organization, referred to as a data partner, are identifiable personal data and within the scope of the Data Protection Act 2018 [19]. As such, we wanted to ensure the team undertaking the data mapping never had access to the underlying data and only operated on metadata that were outside of the scope of data governance regulations. Throughout the process, the only people to have access to or process personal data were the data partners. Such an approach was low risk and ensured privacy-by-design and data minimization principles were strictly followed.

Two software packages were created to separate the data conversion from the creation of transformation rules. The first software package was to handle the creation of mapping rules based solely on the metadata. This tool is called Carrot-Mapper [17,20]. The second software package was designed to reside within the data partner’s network and use the rules to convert the data. This tool was called Carrot-CDM [18]. This approach defines two separate processes: one process to create the rules for mapping and a separate process that takes those rules and applies them to the data.

### Reuse and Reproducibility

FAIR principles seek to ensure the required metadata are captured across the 4 categories of FAIR. Our desire is to embed these same principles into the design of Carrot such that the mappings generated were also FAIR. A key requirement of the software is that mappings from one dataset can assist in the mapping of another, reducing wasted effort. For example, significant work was undertaken in the United Kingdom to standardize questionnaires in response to COVID-19 that were used across many national cohorts [21], that way many different research cohorts agreed to use the same questionnaire rather than each creating their own to gather data from their participants related to COVID-19. Therefore, we wanted to ensure that once the questionnaire had been mapped for one data partner, it can be instantly applied to other datasets. Over time the efficiency can be increased as the mapping rules from previous work can be used in new work.

Data curation to a new standard could be considered an art form, as each individual undertaking such an activity may do so in a different way. As an artist may have a signature brush stroke, it is also true that data engineers will have their preference for how to convert data to OMOP. Therefore, to promote reproducibility our key principle was to minimize individual decision-making and the development of a standardized pipeline that produces the same results after each execution. That process was supported by features designed to provide feedback to the user regarding any curation issues compared with previously mapped datasets (see Mapping Standardization Tools section below).

### Process Architecture

#### Overview

The mapping rule generation web application Carrot-Mapper is built using the Django Python web framework (Django Software Foundation), with the React JavaScript library for user interfaces, and PostgreSQL database. These are supported by serverless Azure Functions written in Python for asynchronous processing of the large amounts of data ingested with each new file upload. The Carrot-Mapper web application automatically deploys via GitHub Actions to Microsoft Azure infrastructure with each new release.

**Figure 1:**
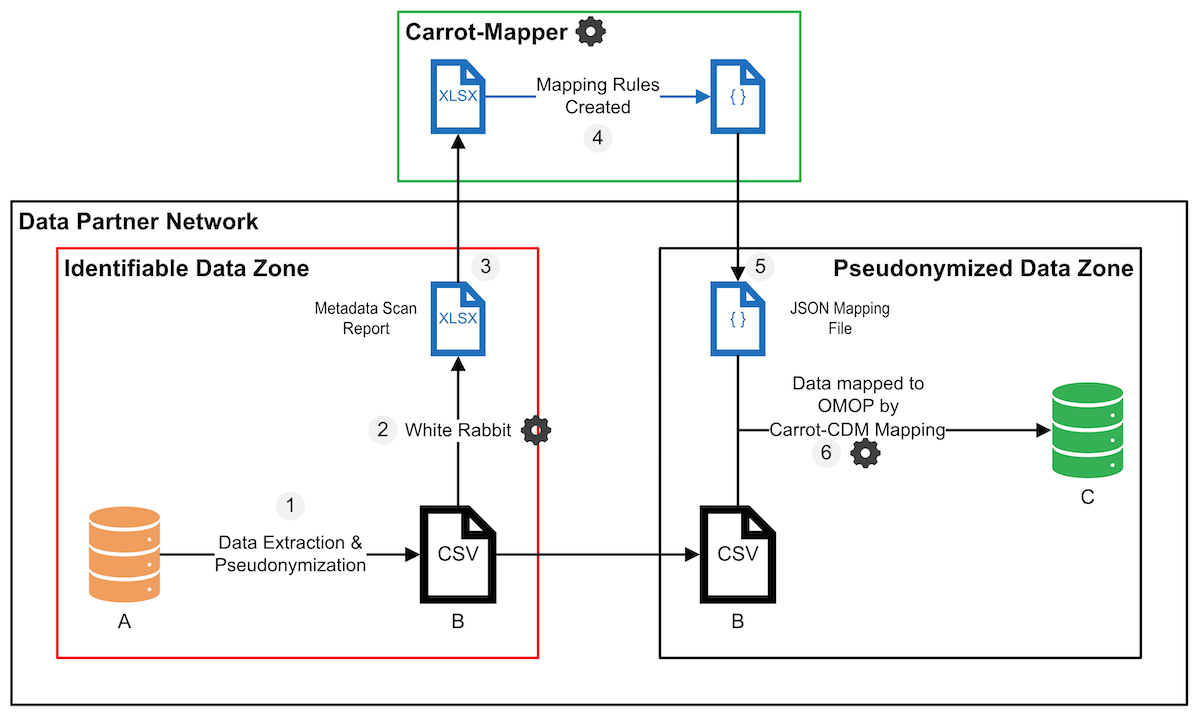
Carrot pipeline overview. (A) The raw, identifiable dataset. (B) Deidentified data. (C) The OMOP standardized deidentified dataset. OMOP: Observational Medical Outcomes Partnership.

The minimal Carrot pipeline consists of 6 steps that are illustrated in Figure 1: (1) preprocess data to remove identifiable data and apply the standards in [22], (2) generate metadata report via White Rabbit [6], (3) transfer metadata file to Carrot infrastructure, (4) generate rules using Carrot-Mapper, (5) transfer rules to system housing data, and (6) use Carrot-CDM to apply generated rules to transform pseudonymized data to OMOP standard.

Note that Carrot-Mapper and Carrot-CDM handle steps 4 and 6, respectively, of the above pipeline. The formats of the metadata report and mapping rules file are open, ensuring that data partners have the option to implement their own drop-in solutions instead of one or the other software tools to fit their own infrastructure if desired. The Carrot team runs a central deployment of Carrot-Mapper, which can be used by multiple projects. A private instance of Carrot-Mapper can also be run within the data partner’s infrastructure, although this reduces the utility of the system as it cannot draw on existing mapping rules generated for previous datasets and nullifies some of the benefits of reduced governance requirements. We ask all data partners to check the metadata and the contents of the scan reports to validate that no identifiable or personal data leaves the data partner’s network at any time.

#### Minimizing Input From Data Partners (Step 1)

Each data partner remains responsible for the management of their data, including generating a pseudonymized and deidentified extract. There are some preprocessing steps that they must perform to use the tool, but we have minimized the workload on each data partner. In order to simplify the later steps of conversion to OMOP, we also ask the data partner to follow the steps at [22] under “Data Preparation,” namely, presenting the data in a series of CSV files, with one CSV containing demographic data, all measurements in the metric system, dates and date times in the ISO-8601 format, digit grouping symbols removed, decimal numbers rounded to a maximum of 2 decimal places, CSV files encoded with UTF-8 and Unix and Linux line endings; and CSV file names limited to 30 characters.

The data partners do have a vital role in ensuring their data is represented correctly and all local insight is maintained during the process. What we have sought to minimize at this stage is the involvement of technical teams and to remove that burden.

#### Generating Metadata (Step 2)

The pseudonymized dataset held within the data partner’s infrastructure (as generated by step 1 of the above 6-step process) is profiled by the open-source White Rabbit [6] data profiling tool, resulting in a metadata file known as a scan report. Alternatively, the scan report can be generated by hand or by other tools, so long as the resulting scan report file (an Excel file containing multiple sheets) has the correct structure.

The scan report details the tables and fields of the dataset, along with values present in each field. The Carrot data standards [22] instruct the data partner in the correct setup of the White Rabbit tool to ensure identifying data is not inadvertently present in the scan report. This includes setting the “Minimum Cell Count” to at least 5 to reduce the probability of possibly reidentifying conditions being included. The scan report should then be checked to remove any remaining data values that could be deemed confidential or sensitive.

A data dictionary may be optionally supplied. This can provide (1) vocabularies associated with certain fields and (2) descriptions of values when the field names and values are not self-explanatory. As an example, it would be possible to specify that a column contains SNOMED values. As the system is using the contents of the Athena system for OMOP, 18 existing vocabularies (eg, SNOMED-CT [23], ICD v9 [24], and Healthcare Common Procedure Coding System [25]; Multimedia Appendix 1) can be specified. The structure of the data dictionary is defined on the Carrot standards page [22].

Importantly these scan reports can also be used by the data partner to register the datasets within publicly available metadata repositories, such as the Health Data Research Gateway [26]. In doing so, we continue our theme of reducing the burden and ensuring one process can result in many benefits.

#### Uploading Scan Report and Data Dictionary Files (Step 3)

Both the scan report and optional data dictionary files are then transferred to the OMOP experts. The OMOP experts upload the scan report file and optional data dictionary file to Carrot-Mapper via a web form. This creates a new scan report instance within the system, with the user able to specify accompanying settings such as the project and dataset to which the scan report belongs, and the visibility and access to the scan report for other users. See the Users and Projects section below.

Upon upload of the scan report file (which can take a few minutes to process large and complex datasets), the Carrot-Mapper tool records all tables, fields, and values within the scan report file. This enables users to visually navigate the structure of the dataset within the mapping tool for the purposes of manual mapping (below) via the browser.

Each scan report has a status field that can be used to track the progress of the scan report upload and the manual mapping process, using the statuses “Upload in Progress,” “Upload Complete,” “Upload Failed,” “Mapping 0%,” “Mapping 25%,” “Mapping 75%,” “Mapping Complete,” and “Blocked.” This status field is automatically updated and can also be set manually from the main scan reports list page, for example, to progress through the stages from “Mapping 0%” to “Mapping Complete.”

The centralized system allows previous mapping rules to be reused. For example, assume a field “Sex” is provided with the value “M,” and this has previously been manually mapped to the OMOP concept 8507 “Male” in one scan R=report. In the case where a later scan report is uploaded with another field “Gender” and the value “M,” the system automatically applies the previous mapping rule to this value in this field in the new scan report. In this way, the utility of the system increases over time, as mapping rules are saved, and the manual effort required to generate mapping rules is reduced. This is particularly the case where a new scan report is supplied that describes a dataset that has been previously partially mapped, or where multiple datasets adhere to a shared standard. Only mappings from scan reports marked with the status “Mapping Complete” are eligible to be considered for reuse. This mechanism allows trusted and verified rules to be replicated, while works in progress are not mined for rules to apply to new scan reports. This mapping reuse algorithm runs at the time when a scan report file is first uploaded. The limitation is that it can only reuse rules when the column name is an exact match and the value is also an exact match. It has no intelligent processes to either translate from languages (such as Spanish to English) or to auto-correct for potential spelling mistakes (“Gendar” to “Gender”).

Additionally during this initial upload process, there is the automated mapping from recognized vocabularies. In the case where an entry in the data dictionary indicates that a field is encoded in a recognized vocabulary, the system will automatically create mappings to the (possibly multiple) standard and valid OMOP concept codes associated with each source code. Some scan reports can contain thousands of unique values in such fields, and as such, this feature can save days of repetitive and error-prone work for OMOP experts.

Throughout this stage, it is always clear how a rule was generated, whether it was manual, a reuse of an existing rule, or using the OMOP in-built relationships (such as mapping ICD to SNOMED). The interfaces of Carrot indicate this as M for manual, R for reuse, and V for vocabulary-based rule generation.

#### Generating Rules (Step 4)

Carrot-Mapper has been created as a web-based tool to assist in the generation of rules. A core reason for establishing a central tool for creating rules is that they can then be reused across projects and datasets. However, the requirement that Carrot-Mapper support rule reuse, and thus mappings related to multiple datasets be stored in a single location, also necessitates user authentication and granular permissions to control access to uploaded data (see Users and Projects section below for more details).

Once a scan report file has been uploaded and processed, manual mapping can proceed. The OMOP expert can navigate the contents of the scan report, organized in a hierarchical manner. The system allows the user to select a field or value and mark it with the desired target OMOP concept codes, as well as remove any incorrect codes (including reviewing those generated through vocabulary lookup or mapping rule reuse in step 3 above). At any stage in the mapping process, the user can see a summary view of the mapping rules currently extant and review, discuss, and remove any mapping rules. Once a user is satisfied that they have mapped all of the required fields and values, they can set the scan report status as “Mapping Complete,” which also makes the mapping rules defined in that scan report available for reuse by other scan reports. Mapping rules can be exported in CSV format (for human readability and review) or in JSON format for ingestion into the Carrot-CDM ETL tool at the next stage of the pipeline.

#### Mapping Rule Generation

Rules are generated using internal logic to minimize the work required from a user who wishes to add a new rule. Each table must have one field identified by the user as a “Person ID” field and one field as “Date Event,” which can be defined via the web application. For every field and value, the user can input the target OMOP concept ID. The web application handles this by looking up the OMOP ID in the OMOP database to access the OMOP term. This term is then mapped to a valid term and the related domain. These data points (valid term, domain, person ID, and date event) are sufficient to define a mapping rule. This feature removes some of the technicalities of OMOP from view, allowing the user to focus purely on the most accurate OMOP representation of the data.

#### Transfer Mapping Rules to Data Partner Network (Step 5)

The mapping rules JSON file, as exported in the previous step, is transferred into the data partner’s network. These are the only data that enter the data partner’s network.

#### Data Transformation (Step 6)

Within the data partner’s infrastructure, the data partner sets up a machine with the Carrot-CDM tool installed. The Carrot-CDM package is an ETL tool to convert a dataset to OMOP using supplied rules. Built with Python, the package can be readily installed in the environment hosting the dataset. The task of Carrot-CDM is to convert the CSV files containing the dataset into an OMOP dataset, based upon the mapping rules generated by Carrot-Mapper.

Carrot-CDM operates by constructing a CDM object, containing a number of tables representing the tables or domains used in the OMOP schema. At present, these tables are a subset of all of those defined by OMOP, due to the priorities of the datasets and pipelines that have used Carrot until now. Once the CDM has been appropriately constructed, then conversion of the contents of the dataset can proceed. The OMOP mapping experts send the mapping rules in JSON format to the data partner, and the data partner loads the mapping rules file into the machine. The data partner also copies their pseudonymized dataset into the machine. Carrot-CDM is used to transform the dataset to the OMOP standard using the mapping rules provided. Carrot-CDM is platform independent and can be used via either command-line interface or graphical interface, depending on the user’s preference.

Carrot-CDM handles both one-time data transformation and incremental transformation of an expanding dataset as new data is made available, using the same mapping rules as in the one-time case. Carrot-CDM can either monitor for the addition of new data and perform the transformation immediately or run on a schedule to run a transformation of new data added in each period, such as via a server-based scheduler. This means that longitudinal datasets, or those otherwise growing over time, can be efficiently mapped to OMOP. Carrot-CDM supports streaming, enabling essentially infinite datasets to be processed, as working memory is not a limiting factor.

### Data Partner Validation

The data partners clearly hold the vital contextual information to provide assurance that the data mapping is appropriate and correct. Therefore, through steps 1, 2, and 6 (Figure 1) of the process, the data partner is consulted and many questions and clarifications are sought between both parties. The metadata extraction and initial upload can often highlight some discrepancies in data format and can highlight where the data dictionary does not exactly match all of the data picked up in the scan report. Therefore, the recommended process is for a highly collaborative and iterative process whereby the Carrot team has the insight in the process of curating data to OMOP, while the data partner has the insight and understanding of their data.

### Users and Projects

Carrot-Mapper assigns user accounts to individuals and has an internal data model to organize dataset mappings and administrate access to them. These structures are projects, datasets, and scan reports. The basic unit is the scan report, formed from a single uploaded scan report file generated by the White Rabbit tool. Each scan report is a member of a single dataset. Multiple scan reports can be organized into a single dataset to represent successive iterations of a single dataset, such as with the addition of new tables, fields, or values. The usage pattern of datasets and scan reports is left to the user’s choice.

At the highest level, projects represent the abstract notion of a view of a collection of datasets that are of interest to a particular group of users. A user must be a member of a project to access the datasets associated with that project. A many-to-many relationship means that datasets can be present in multiple projects, and it is sufficient that a user is a member of any related project to access a given dataset.

Assuming that a user has access to a given project, user access can then be further controlled on the level of datasets and individual scan reports. Permission to view datasets and scan reports is controlled first by setting their visibility as either “Public” or “Restricted,” and then more granular viewing, editing, and administration operations can be controlled at the per-user level.

### Mapping Standardization Tools

An additional internal tool within Carrot-Mapper highlights related OMOP concepts that are the target of mappings in the dataset corpus. This is provided as feedback to the mapping team to highlight misaligned target concepts (ie, those which are descendants or ancestors of other target concepts in the hierarchical OMOP structure) and encourage iterative convergence on a standardized OMOP vocabulary. Users are free to ignore this guidance if they choose, but the presentation of the potential inconsistencies places this information in the user’s hands without extra effort.

### Management

Carrot-Mapper presents users with a dashboard to show relevant statistics such as the total number of scan reports processed and mapping rules generated, grouped by data partner, and to track the progress of scan reports from the upload stage through to the completion of the mapping process.

User access to datasets and scan reports is configurable via dedicated administration pages for each, placing the user control into the hands of the dataset administrators to reduce reliance on the central Carrot-Mapper team.

### Ethical Considerations

Research ethics approval was not required for this project as each data partner maintains their own governance and ethics for the original research studies. Anyone requiring access to the platform to perform research needs to apply for their own ethics approval. The data partners who use the Carrot system and methods in this paper will need to have the required ethics in place for the collection, storage, and use of data in place.

## Results

As of July 2024, the installation of Carrot-Mapper has been used to generate mappings for 39 scan reports to the OMOP standard. There are 129 dataset objects across 13 projects. A total of 60,269 mappings have been generated, through manual means (n=6159), automatic (n=45,316), and reuse between scan reports (n=8794). These numbers are only for those scan reports marked as “Mapping Complete,” indicating that they have been accepted as correct by the users, and their associated mappings are now available for reuse by new datasets. More (n=92,071) mappings are currently in progress without having been marked as archived or as completed.

This use of Carrot tools has been adopted by a number of projects, including COVID-Curated and Open Analysis and Research Platform (CO-CONNECT; see below), Alleviate [27], Defining Mechanisms Shared Across Multi-organ Fibrotic Disease to Prevent the Development of Long Term Multimorbidity (DEMISTIFI),

Mother and Infant Research Electronic Data Analysis (MIREDA), the National Institute for Health and Care Research Nottingham Biomedical Research Center, and most recently the East Midlands Secure Data Environment. Carrot is therefore an integral part of ongoing efforts by several organizations to standardize health data to the OMOP standard. Since Carrot-Mapper can be deployed by any user, with a separate database, it is not possible for the authors to accurately gauge the use of Carrot-Mapper beyond the centrally installed system deployed by the Carrot team.

Most mappings (over 75%) were automatically generated from vocabularies, indicating the importance and time-saving nature of the automated mappings feature. Existing mappings that have been verified are automatically reused on newly uploaded scan reports where they match, reducing wasteful replicated effort. While the proportion of mapping that is from reuse is currently low (less than 15%), we anticipate (and have seen even thus far) that as the number of mapped values increases, and iterations of the same dataset are progressively remapped over time, the number of reused mappings will rise compared with the number of manually added mappings. Even at this early stage, nearly 60% of the mappings that are not automatically generated from known vocabularies are from reuse.

## Discussion

### Principal Findings

Accessing and combining multiple datasets can be handled in a variety of ways. Principally, the choice of whether to standardize vocabularies then drives the choices available in further steps in any processing pipeline. Leaving datasets in their original formats requires later processing steps to understand and handle the heterogeneity in the underlying data sources. While this can be handled on a case-by-case basis for small numbers of datasets, at scale, this requires the creation and maintenance of a large number of connectors and middleware given the plethora of possible data sources. This is the approach taken by systems with a limited number of data sources such as OpenSAFELY [28] but is not an approach that is sustainable for many smaller datasets and data partners.

Converting datasets to a standardized vocabulary reduces the heterogeneity of the datasets at an earlier step in the pipeline, simplifying later processing steps and reducing the dataset-specific knowledge required by users of the data. However, it places the onus on formulating a consistent and correct mapping to the standard vocabulary. It is also not possible to cover every use case with a single standardized vocabulary, so some pipelines may be best served by using the raw data, while other use cases may lend themselves to a specific standardized vocabulary that is inappropriate for other uses.

In choosing a common standard vocabulary, a number of competing factors must be considered, including the suitability of the vocabulary to represent the original datasets, ease of conversion, availability of required expertise and tooling, and adoption by others. A wealth of standard vocabulary candidates exist, including OpenEHR, OMOP, and PCORnet [29].

Converting a dataset to a common standard can sometimes be partially automated—in the case of OMOP, many research ontologies such as SNOMED can be mapped directly—but in almost all cases this conversion requires a human element to decide the most appropriate mapping. This brings with it privacy and data protection implications if the process of manual mapping relies upon providing access to the dataset. The EHDEN program funds the conversion of datasets to the OMOP standard by using approved organizations to perform the conversion. This relies on governance assurances to ensure that only trusted and approved individuals are granted access to the dataset under conversion. This can be a relatively quick method for converting high-priority datasets to the OMOP standard but will not enable the conversion of lower-priority datasets given the competitive nature of the process.

The Carrot tools take a different approach, by only extracting to a central system the metadata required from the dataset to formulate a mapping. In this way, experts in OMOP conversion with access to the central Carrot system can create a mapping from the original dataset to the OMOP standard without ever having access to either identifiable or pseudonymized and anonymized data. This removes some of the governance requirements. In addition, it allows the creation of a corpus of mapping rules that can be shared and reused between datasets, and compared and aligned over time. Finally, Carrot can automate much of the mappings required from recognized standard vocabularies, reducing costs and opportunities for error.

The generation of rules by automated processes does have the ability to create more harm than good, as a human has to check and correct any potential errors. The use of automation in Carrot is limited to two scenarios. The first is where a concept has already been formally mapped by the OHDSI vocabulary from a nonstandard to standard concept. The second is where a previously human-approved mapping can be reused because the column and values exactly match. The user is presented with these and can see which rules were mapped from the vocabulary (marked with a V) and from reusing existing rules (marked with an R). We are currently gathering more evidence on the utility of these automated approaches and whether other automation is useful, such as the adoption of large language models.

The novel nature of the metadata access required by Carrot tools has created some additional governance issues, since many organizations did not have existing data access processes that were used to process lower risk metadata. In the use-case of CO-CONNECT, this required an extensive collaborative effort between our team and data partner organizations to satisfy them with the nature of the requests and to create new processes to enable them. This delayed some of the mapping work but is also an important output from the wider CO-CONNECT project.

### Phenotype Generation

Bringing together the metadata from multiple datasets and their mappings to the OMOP standard enables leveraging the combined data to build phenotypes. For example, all fields and values across all datasets that are mapped to the OMOP concept code 317009 for asthma can be queried. In future work, we plan to provide a publicly available tool to present this information, allowing interested parties to interrogate the source values that map to a given OMOP concept code. This is valuable for understanding the varying ways in which this data is captured across datasets and allows external validation of the mappings generated by the central team.

### Pipeline Integration

The Carrot tools have been built in such a way to be integrated into wider pipelines, as demonstrated in the CO-CONNECT project. The separation of the tasks into separate tools based on their requirements for human intervention provides a natural fit for integration into semiautomated pipelines. Work in this area continues, such as future extensions to support the postprocessing of OMOP data to encode relationships, work to further develop the JSON mapping rules file specification to fit common standards, and support of data-profiling tools in addition to White Rabbit.

### Limitations

The Carrot tools remain under active development to extend and improve their functionality. In particular, work remains to monitor and handle the continual changes made to the OMOP vocabulary to provide reproducibility and transparency to the processes. The priorities of the initial use cases shaped the development process, in particular regarding support for OMOP tables. Further work is ongoing to widen the target OMOP tables the Carrot tools can support. The work presented has not run different methods in parallel; therefore, we make no claim over whether this approach is better, faster, or more efficient or uses less computing resources than others. Where data governance and protocols would allow such a comparison could be useful to evidence the benefits of the system. What we have sought to lay out is a protocol, supported by software, that was driven by the underlying constraints and challenges experienced.

The matching algorithm for reusing mappings across datasets is relatively simple, and ripe for further refinement as well as further user customizability. That is because even a small spelling mistake would prevent a match from being found. Additionally, Carrot cannot undertake any natural language processing, nor can it translate between languages.

Finally, further development remains to provide greater user ease, particularly around the self-administration of user accounts and streamlining the user journey to eliminate known areas that could be further automated.

While reducing some aspects of technical knowledge that are required by users (eg, removing the need for SQL knowledge typically required in most other workflows and automating the details of which OMOP concept codes relate to which target OMOP table), Carrot does still rely on an expert understanding of OMOP to be used to greatest effect. In this regard, it is to enhance rather than replace the capabilities of human curators and makes no claim to be able to process full datasets from end to end without human oversight and intervention. There is still a need for clinical specialties (eg, chronic pain) to agree on correct and consistent usage of terms to improve the interoperability of disparate datasets.

### Conclusions

We have presented the Carrot tools, namely the Carrot-Mapper and Carrot-CDM software tools. These enable expert or novice curators to transform health datasets to the OMOP standard with access only to metadata, rather than the potentially sensitive data. This has the potential to radically reduce the governance work and cost required to perform this transformation, without sacrificing the expertise that can be brought by external curators.

Carrot-Mapper contains functionality to specifically reduce the repeated work required to map multiple datasets by reusing mappings between datasets. It further contains tools that can guide curators toward standardized mappings over time, reducing the dependence upon individual curators’ different approaches to the preferred terminologies to represent data. All of this is achieved without ever exposing the sensitive data to the external team with OMOP knowledge.

Carrot-CDM is a robust and scalable ETL tool that can run within the data partner’s infrastructure, completing the journey to an OMOP dataset without data movement beyond the data partner’s boundaries, nor requiring external users to be granted access. In combination, the Carrot tools provide a means for OMOP experts to convert health care datasets to OMOP in a standardized manner, with reduced governance requirements compared with the existing tools available.

## Appendix

Multimedia Appendix 1 Details of the vocabularies that are used in Carrot-Mapper as default.

## Abbreviations

CDM: common data model
CO-CONNECT: COVID-Curated and Open Analysis and Research Platform
DEMISTIFI: Defining Mechanisms Shared Across Multi-Organ Fibrotic Disease to Prevent the Development of Long Term Multimorbidity
EHDEN: European Health Care Data and Evidence Network
ETL: extract, transform, and load
FAIR: Findable, Accessible, Interoperable, and Reusable
GDPR: General Data Protection Regulation
ICD: International Classification of Diseases
OHDSI: Observational Health Data Sciences and Informatics
OMOP: Observational Medical Outcomes Partnership
SNOMED: Systematized Nomenclature of Medicine
SNOMED-CT: Systematized Nomenclature of Medicine Clinical Terms

## Data Availability

There is no data to make available, but all the Carrot software is available on an open-source license.
