# Supplementary material for: Conversion of Sensitive Data to the Observational Medical Outcomes Partnership Common Data Model: Protocol for the Development and Use of Carrot
Source: JMIR Res Protoc. 2025 Apr 2;14:e60917. doi: 10.2196/60917 (PMC12004012; doi:10.2196/60917)
Supplement: Multimedia Appendix 1 [file resprot_v14i1e60917_app1.docx]

# Appendix 1

List of vocabularies:

- CO-CONNECT
- CO-CONNECT MIABIS
- CO-CONNECT TWINS
- HCPCS
- HES Specialty
- ICD10
- ICD10CM
- ICD9CM
- LOINC
- OMOP Extension
- OPCS4
- PPI
- Read
- SNOMED
- SPL
- UB04 Pri Typ of Adm
- UB04 Typ bill
- UK Biobank
